# Conventional type 1 dendritic cells protect against age-related adipose tissue dysfunction and obesity

**DOI:** 10.1038/s41423-021-00812-7

**Published:** 2022-01-04

**Authors:** Elena Hernández-García, Francisco J. Cueto, Emma C. L. Cook, Ana Redondo-Urzainqui, Sara Charro-Zanca, Iñaki Robles-Vera, Ruth Conde-Garrosa, Ivana Nikolić, Guadalupe Sabio, David Sancho, Salvador Iborra

**Affiliations:** 1 Department of Immunology, Ophthalmology and ENT, School of Medicine, Universidad Complutense de Madrid, Madrid, 28040 Spain; 2 Centro Nacional de Investigaciones Cardiovasculares (CNIC), Madrid, 28029 Spain

**Keywords:** Dendritic Cells, NKT, Obesity, ageing, FLT3L, Conventional dendritic cells, Chronic inflammation

## Abstract

Conventional dendritic cells (cDCs) scan and integrate environmental cues in almost every tissue, including exogenous metabolic signals. While cDCs are critical in maintaining immune balance, their role in preserving energy homeostasis is unclear. Here, we showed that Batf3-deficient mice lacking conventional type 1 DCs (cDC1s) had increased body weight and adiposity during aging. This led to impaired energy expenditure and glucose tolerance, insulin resistance, dyslipidemia, and liver steatosis. cDC1 deficiency caused adipose tissue inflammation that was preceded by a paucity of NK1.1^+^ invariant NKT (iNKT) cells. Accordingly, among antigen-presenting cells, cDC1s exhibited notable induction of IFN-γ production by iNKT cells, which plays a metabolically protective role in lean adipose tissue. Flt3L treatment, which expands the dendritic cell (DC) compartment, mitigated diet-induced obesity and hyperlipidemia in a Batf3-dependent manner. This effect was partially mediated by NK1.1^+^ cells. These results reveal a new critical role for the cDC1-iNKT cell axis in the regulation of adipose tissue homeostasis.

## Introduction

Natural selection pressures act against high adiposity due to the risk of predation while also disfavoring low levels of adiposity because of the risk of starvation and inability to mount energetically demanding immune responses against diseases and pathogens [1]. The integrated immunometabolic response (IIMR) senses the general metabolic status of the body and, more specifically, environmental metabolic stressors, such as prolonged fasting (reviewed in [2]). The IIMR involves neuronal (sympathetic and sensory innervation) and humoral (insulin, ghrelin, leptin, etc.) signaling between the hypothalamus and peripheral tissues. Obesity underlies many comorbidities, such as cardiovascular diseases, metabolic syndrome, liver steatosis, and cancer. It is also associated with a high incidence of chronic autoimmune and inflammation-related pathologies, such as type 2 diabetes mellitus, nonalcoholic fatty liver disease, osteoarthritis, and rheumatoid arthritis. In addition to being a nutrient depository, adipose tissue (AT) actively modulates whole-body metabolism by secreting cytokines that alter tissue microenvironments. AT expansion leads to adipocyte hypoxia, apoptosis, and cell stress, ultimately resulting in the expression of chemoattractant molecules and infiltration of inflammatory cells. Chronic low-grade inflammation in white adipose tissue (WAT) is a common link among obesity, insulin resistance (IR), and metabolic disease [3]. Aging is also associated with an increase in abdominal WAT [4, 5], which significantly augments IR. In addition to lifestyle changes in elderly people, which may cause a chronic state of positive energy balance [6], immunosenescence may cause deregulation of immune infiltrates, contributing to age-associated obesity.

Macrophages and dendritic cells (DCs) are well equipped to sense environmental cues and play dominant roles in tissue homeostasis and inflammation. Macrophages comprise the most abundant leukocyte population in the adipose stromal fraction, and their number increases with increasing adiposity in both mice and humans [7, 8]. The tyrosine kinase receptor Flt3 and its ligand (Flt3L) regulate DC development in the steady state [9]. Flt3l^KO^ mice, which are devoid of DCs, gain less weight than wild-type mice when fed a high-fat diet (HFD) and have reduced numbers of macrophages in their WAT [10], suggesting that DCs play a pathogenic role during overnutrition. However, Flt3l^KO^ mice also have defects in early hematopoietic progenitors, NK cells [11], and Group 2 and 3 ILCs [12], so the observed effect may not only rely on DCs. Moreover, DCs comprise three main subsets, plasmacytoid DCs (pDCs), myeloid/conventional DCs, which are further  subdivided in cDC1s and cDC2s, with different ontogenies, surface markers, localizations, and immunological functions. The cDC1 lineage, whose development depends on basic leucine zipper ATF-Like transcription factor 3 (Batf3) [13–16], is defined by its selective expression of the chemokine receptor XCR1 [17] and is endowed with a high intrinsic capacity to cross‐present antigens via MHC class I and the abilities to activate CD8^+^ T cells and promote T helper type 1 (Th1) and natural killer cell responses through IL‐12. The secretion of XCL1, the unique known ligand of XCR1, by memory T cells, NK cells [18], and NKT [19] cells allows these cells to interact with cDC1s. These immune populations are characterized by the secretion of interferon-γ (IFN-γ) and  tumor necrosis factor (TNF-*α*). These cytokines, along with other proinflammatory signals, enhance the accumulation of monocyte-derived macrophages and “M1” polarization into proinflammatory macrophages [20, 21].

Here, we found that cDC1 infiltration within WAT relied on the general metabolic status of the body and set out to explore the function of cDC1s in obesity. We found that Batf3 deficiency increased body weight gain and adiposity in middle-aged mice fed a standard chow diet (SD). Batf3-deficient mice exhibited reduced energy expenditure and altered oxygen consumption. M1-like macrophages accumulated in mice deficient in cDC1s, correlating with increased TNF*α* expression in the tissue, impaired glucose tolerance, dyslipidemia, and liver alterations. Analysis of immune infiltrates in the WAT of Batf3-deficient mice before the onset of the obese phenotype revealed impaired WAT infiltration by NKT and NK cells. Notably, administration of systemic sFLT3L restrained high-fat diet-induced obesity (DIO) through the expansion of cDC1s, ameliorating hepatomegaly and hyperlipidemia. Thus, our work identifies cDC1s as a protective DC subset that prevents the onset and evolution of age- and diet-related obesity and associated morbidities.

## Results

### cDC1 abundance in eWAT varies with the nutritional status and correlates with weight gain

**Fig. 1:**
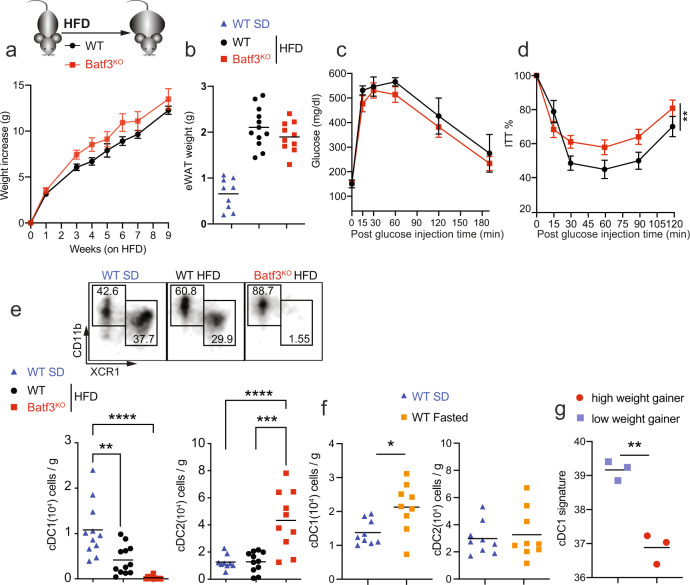
Diet-induced obesity in Batf3-deficient mice and cDC1 abundance in eWAT during nutritional status variation. **a**–**e** WT and Batf3^KO^ mice were fed an HFD for 9 weeks. **a** Weight gain (*n* = 9), **b** weight of epididymal white adipose tissue (eWAT), and **c** glucose levels in serum samples during a glucose tolerance test (GTT; WT *n* = 6, Batf3^KO^ *n* = 11). **d** Percentages of baseline glucose levels in mice of the indicated genotypes during an insulin tolerance test (ITT; WT *n* = 6, Batf3^KO^ *n* = 11). **e** Upper panels: representative dot plots of CD11b^+^ (cDC2s) and XCR1^+^ (cDC1s) DCs in the stromal vascular fraction (SVF) of eWAT (**b**), lower panels: quantification of cDC1s and cDC2s per gram of eWAT. **f** Quantification of cDC1s and cDC2s per gram of eWAT in WT mice starved for 36 h. **g** cDC1 signature scores for the epididymal fat of inbred mice that would subsequently become high or low weight gainers after exposure to a high-fat diet (GSE4692 [27]). Significance was assessed by an unpaired two-tailed Student’s *t*-test. *P < 0.05; **P < 0.01; ***P < 0.001; ****P < 0.0001. Each point represents a biological replicate. (**a**, **c**, and **d**) Data are presented as the mean ± SEM, and the same mice were measured repeatedly.

Batf3-dependent cDC1s are key in priming proinflammatory Th1 responses and activating CD8^+^ T cells by cross-priming in different settings [13, 22–24]. Since obesity progresses with an increase in IFN-γ-producing cells in adipose tissue [25], we hypothesized that Batf3 deficiency may impact DIO. However, we found no difference in body weight gain (Fig. 1a) or epididymal WAT (eWAT) weight (Fig. 1b) between Batf3-deficient (Batf3^KO^) mice and WT control mice fed an HFD. Batf3 deficiency did not affect fasting glucose levels or glucose disposal during a glucose tolerance test (Fig. 1c). However, Batf3-deficient mice showed higher insulin resistance than their WT counterparts (Fig. 1d). Contrary to what has been shown in other inflammatory settings [14, 26], other Batf paralogs were not able to compensate for Batf3 deficiency in cDC1 development in this setting, since XCR1^+^ or CD103^+^ DCs were almost absent in the stromal vesicular fraction (SVF) of the eWAT of these HFD-fed mice (Figs. 1e and S1a). However, we observed that while the abundance of cDC2s in WT mice did not change significantly upon HFD feeding, cDC1s were less abundant (Fig. 1e), which could partially explain the absence of a Batf3-dependent effect. In contrast, WT mice fasted for 36 h had more cDC1s in their eWAT than those fed an SD *ad libitum* (Fig. 1f), suggesting that cDC1 infiltration in WAT might be promoted by severe starvation, a response to the metabolic status of the mice. We performed a retrospective analysis of expression profiling by an array (GSE4692) to examine the gene expression in the eWAT of low and high weight gainers after 4 weeks of HFD feeding [27]. To investigate the possible association between cDC1 abundance in eWAT and weight gain, we generated a cDC1 score based on the expression levels of *Clec9a*, *Xcr1*, and *Batf3*. Interestingly, low weight gainers displayed a significantly higher cDC1 score than high weight gainers (Figs. 1g and S1b), suggesting that the presence of cDC1s is associated with lower weight gain.

### Batf3 deficiency increases body weight gain and adiposity in mice fed a standard chow diet

**Fig. 2:**
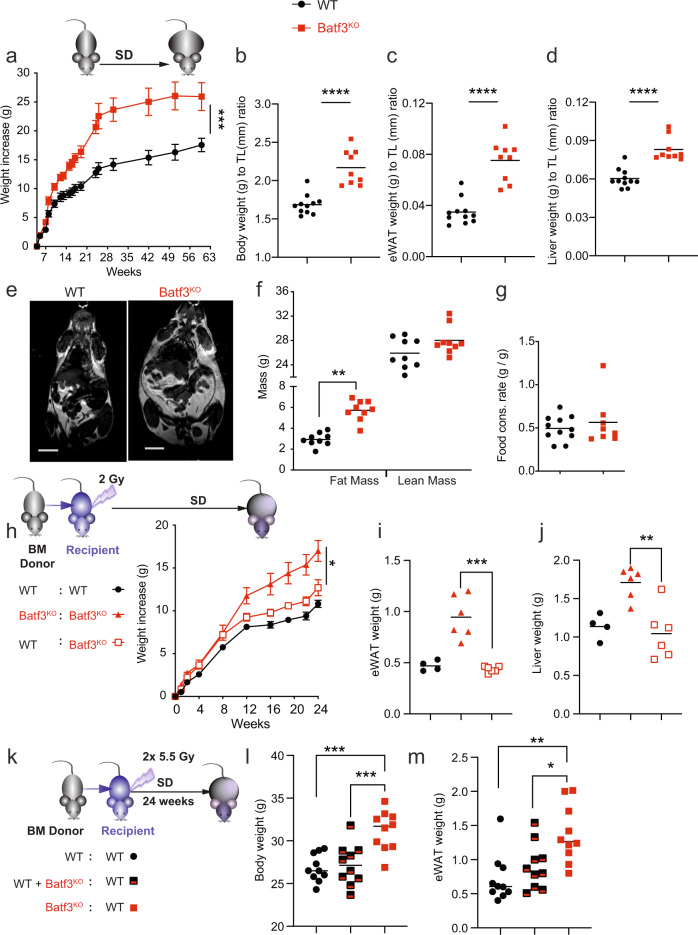
Lack of Batf3 increases body weight gain and adiposity in mice fed a standard chow diet. **a** Body weight gain (*n* = 7) in cohoused mice of the indicated genotypes. Weights normalized to the tibia length (mm) (**b**), eWAT (**c**), and liver (**d**) of mice at 30 weeks of age. **e** Magnetic resonance imaging (MRI) showing body composition and **f** quantification of lean and fat mass among 30-week-old mice of the indicated genotypes. Scale bars, 1 cm. **g** Food consumption rate (g/g). **h** Reconstitution of cDC1s in Batf3-deficient mice and reconstitution of lethally irradiated WT mice with Batf3-deficient BM-derived cells. Mice of the indicated genotypes were either sublethally (**h**–**j**) or lethally irradiated (**k**–**m**) and adoptively transferred with BM cells from WT or Batf3-deficient mice. Weight increase (**h**) or body weight (**l**) was monitored, and eWAT weight (**i**, **m**) and liver weight (**j**) were assessed 24 weeks after irradiation. Significance was assessed by an unpaired two-tailed Student’s *t*-test. *P < 0.05; **P < 0.01; ***P < 0.001; ****P < 0.0001. Each point represents a biological replicate. **a**, **h** The same mice were measured repeatedly. **a**, **h** Data are presented as the mean ± SEM

Aging also disrupts metabolic homeostasis, leading to obesity, visceral adipose tissue accumulation, and inflammation. Interestingly, while cDC2s and macrophages became more abundant with aging (Fig. S1c), as expected [28], the number of Batf3-dependent cDC1s per gram of eWAT did not change. We sought to determine whether cDC1s regulate age-related weight gain in mice fed an SD. Of note, Batf3^KO^ mice showed increased body weight compared with WT mice, with this difference becoming significant around week ten in male mice cohoused immediately after weaning (Fig. 2a), which controlled for the effect of commensal microbiota in these mice, and around week 18 in females (Fig. S1d and S1e). Both female and male Batf3^KO^ mice at week 30 showed increased body weight compared with WT mice (Fig. S1f). Batf3-deficient males at 30 weeks of life had an increased body weight normalized to tibia length (TL) (Fig. 2b), with increased eWAT weight (Fig. 2c, S1g, h) and liver weight to TL ratios (Fig. 2d), compared to cohoused WT mice. Increased body weight, adiposity, and hepatomegaly were still apparent in old Batf3-deficient mice (72 weeks of age) (Fig. S1i–k). Analysis of whole-body composition by magnetic resonance imaging (MRI) showed an increase in fat mass but not in lean mass in Batf3^KO^ mice compared with cohoused WT mice (Fig. 2e, f), which was not due to an increased food consumption rate at this stage (Fig. 2g). These data indicate that Batf3 deficiency promotes fat accumulation and overweight.

Since Batf3 overexpression may inhibit adipogenesis by acting directly on adipocytes [29], we investigated whether the presence or loss of Batf3 in BM-derived cells prevents or promotes weight gain and adiposity, respectively, in this setting. First, we reconstituted cDC1s in sublethally irradiated Batf3^KO^ mice by adoptive transfer of WT bone marrow (BM) cells (Figs. 2h and S1l). Batf3^KO^ mice reconstituted with WT BM gained less weight (Fig. 2h) and had lower eWAT (Fig. 2i) and liver weights (Fig. 2j) than Batf3^KO^ mice reconstituted with Batf3-deficient BM. Second, following lethal irradiation and adoptive transfer of Batf3-deficient BM into WT hosts, we observed increased body weight and adiposity (Fig. 2k–m) in comparison with the transfer of WT BM or a mixture of BM cells from both genotypes. Therefore, our results indicate that Batf3-dependent BM-derived cells, most likely cDC1s, restrain fat mass accumulation.

### Batf3 deficiency reduces energy expenditure and alters oxygen consumption in middle-aged mice

**Fig. 3:**
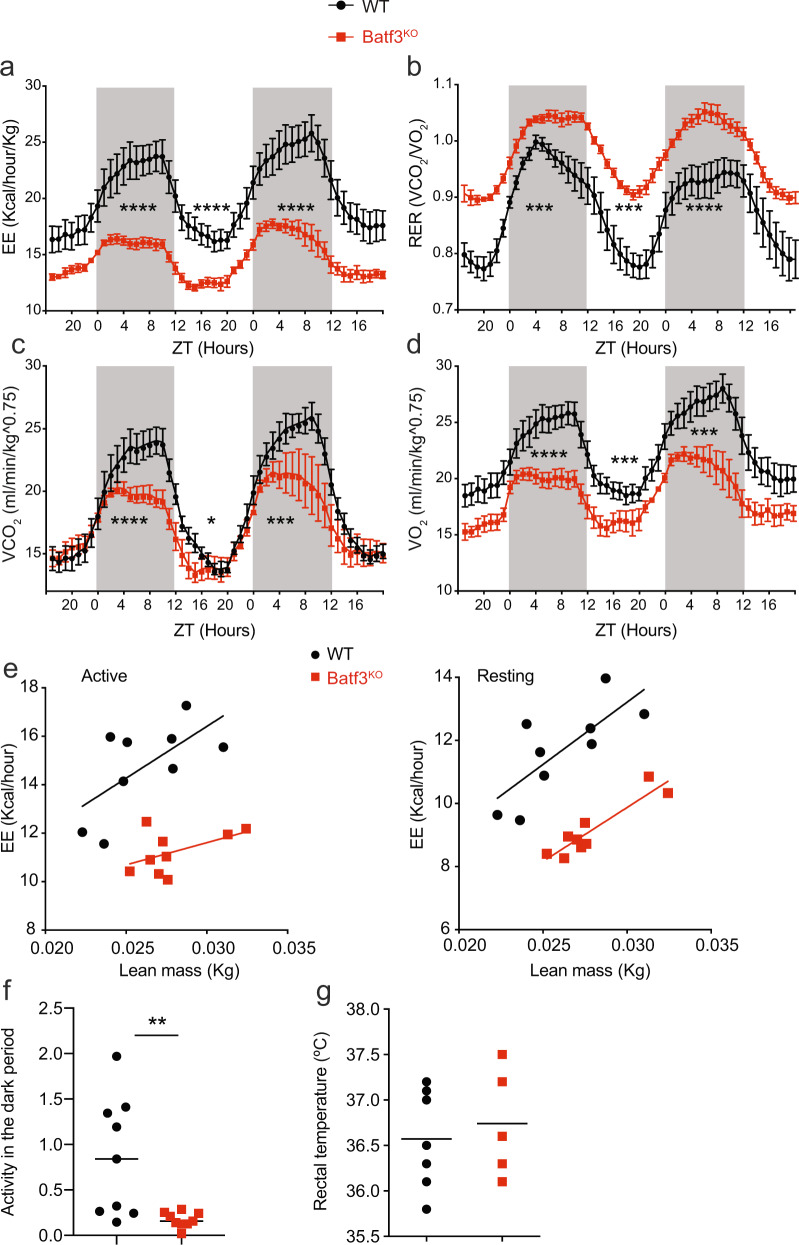
Batf3 deficiency reduces energy expenditure in middle-aged mice and alters oxygen consumption. WT and Batf3^KO^ mice at 30 weeks of age were analyzed in metabolic cages for 48 h (*n* = 7); **a** energy expenditure (EE, Kcal/h/kg), **b** respiratory exchange ratio *(*RER), **c** expired CO_2_ volume (VCO_2_), and **d** consumed O_2_ volume (VO_2_) normalized by body weight (lower) (*n* = 6). **e** Plots representing EE per the whole animal against lean body mass (ANCOVA) in both the active and resting phases. **f** Mean activity in the dark period of mice in metabolic cages for 48 h. **g** Rectal body temperature. Significance was assessed by an unpaired two-tailed Student’s *t*-test. **P < 0.01; ***P < 0.001; ****P < 0.0001. Each point represents a biological replicate. Significance in (**a**), (**b**), (**c**), and (**d**) was determined by comparing the AUCs of the light and dark periods. **a**–**d** Data are presented as the mean ± SEM, and the same mice were measured repeatedly

To investigate the mechanisms by which the lack of Batf3 causes overweight in middle-aged mice, we evaluated whole-body metabolism using metabolic cages. We found a significant decrease in whole-body energy expenditure (EE) in 30-week-old Batf3^KO^ mice fed an SD (Fig. 3a), together with a higher respiratory exchange ratio (RER) (Fig. 3b). This increased RER was due to a modest decrease in expired CO_2_ in the light cycle in Batf3^KO^ mice compared to WT mice (Fig. 3c). This was concomitant with a profound reduction in O_2_ consumption in Batf3-deficient mice during resting conditions (Fig. 3d), suggesting that these mice exhibit a shift in fuel usage from fatty acids to carbohydrates that promotes fat deposition. When plotting the EE of WT and Batf3-deficient mice against their lean body mass, each group was plotted on two separate lines with comparable slopes but intersected the *y*-axis at different heights (Fig. 3e), indicating that there is an effect of genotype on EE regardless of the measured lean mass. In addition, Batf3-deficient mice were less active (Fig. 3f) and consumed less water (Fig. S2a), without obvious differences in rectal (Fig. 3g) or interscapular skin temperature (Fig. S3b). Altogether, these data suggest that Batf3 controls systemic energetics, facilitating fatty acid usage.

### Batf3 deficiency causes adipose tissue inflammation in middle-aged mice

**Fig. 4:**
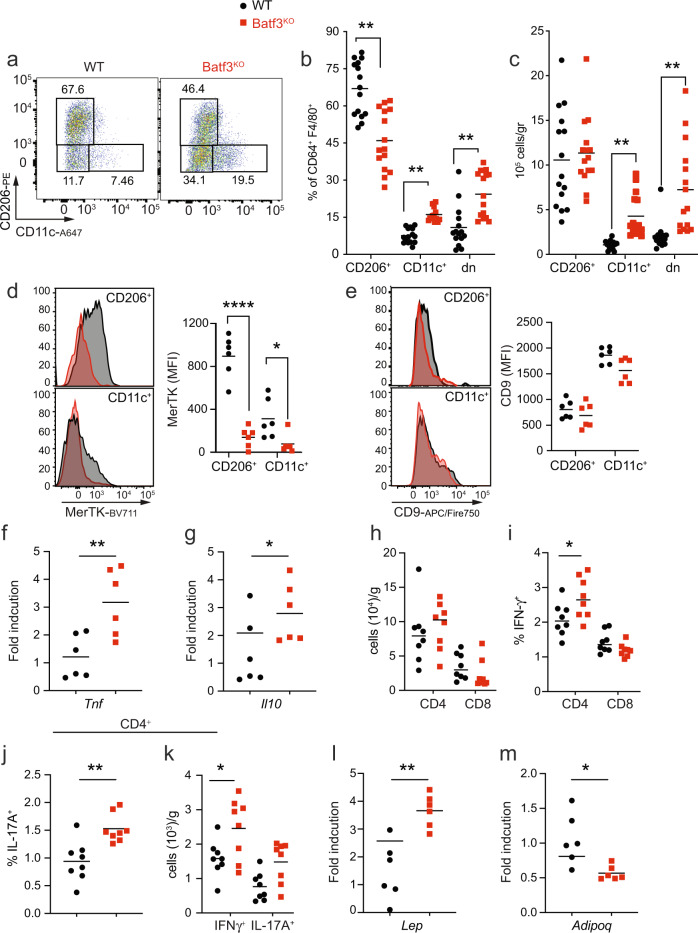
Batf3 deficiency causes adipose tissue inflammation in middle-aged mice. **a** Representative flow cytometry dot plots of CD11c^+^ (M1-like) and CD206^+^ (M2-like) ATMs (CD64^+^F4/80^+^) in the eWAT of 30-week-old WT and Batf3^KO^ mice fed an SD. Analysis of the percentages (**b**), and total amounts (**c**) of CD11c^+^, CD206^+^ and double-negative (dn) ATMs. **d**, **e** Representative flow cytometry histograms and quantification (MFI) of MerTK (**d**) and CD9 (**e**) expression in the indicated ATM populations. Quantification of the eWAT mRNA expression of **f** *Tnf* and **g** *Il-10*. **h** Total numbers of T cells. Percentages of IFN-γ-producing CD4^+^ and CD8^+^ T cells (**i**). Percentages and numbers of IFN-γ and IL-17-producing CD4^+^ T cells (**j**, **k**) upon in vitro stimulation of the SVF of eWAT from mice of the indicated genotypes. Quantification of the eWAT mRNA expression of leptin (**l**) and adiponectin (**m**). Significance was assessed by an unpaired two-tailed Student’s *t*-test. In **d** and **e**, significance was assessed by one-way ANOVA with Sidak’s correction for multiple comparisons. *P < 0.05; **P < 0.01; ****P < 0.0001. Each point represents a biological replicate

Mice lacking DCs have reduced AT macrophage (ATM) numbers [10]. Thus, we investigated the infiltration of adipose tissue by CD11c^+^ classically activated (M1-like) and CD206^+^ alternatively activated (M2) ATMs in middle-aged mice with or without cDC1 deficiency (Fig. 4a). Increased proportions (Fig. 4b) and abundances (Fig. 4c) of M1-like ATMs (CD11c^+^) and CD206^–^CD11c^–^ (dn) cells were observed in the absence of Batf3. Concomitantly, Batf3-deficient mice had a decreased proportion but similar abundance of CD206^+^ ATMs (Fig. 4b, c), suggesting that Batf3 deficiency favors proinflammatory ATMs without affecting homeostatic ATMs. Moreover, both CD11c^+^ and CD206^+^ ATMs from Batf3-deficient mice showed reduced expression of MerTK (Fig. 4d), a hallmark receptor of tissue-resident alternatively activated macrophages that promote apoptotic cell clearance and inflammation resolution. Conversely, CD11c^+^ ATMs from WT and Batf3-deficient mice showed comparable expression of CD9 (Fig. 4e), a marker of proinflammatory lipid-laden ATMs residing within crown-like structures [30]. The accumulation of proinflammatory ATMs found in the absence of Batf3 occurred in parallel with increased mRNA expression of *Tnf* in eWAT (Fig. 4f) and increased *Il10* expression (Fig. 4g). Adipose tissue exhibiting Batf3-dependent inflammation contained comparable numbers of total T cells (Fig. 4h) but mild increases in the proportions and numbers of Th1 (Fig. 4i, k) and Th17 (Fig. 4j, k) cells in the WAT but no increases in IFN-γ-producing CD8^+^ T cells (Fig. 4i). In addition, adipokine production was altered in the WAT of Batf3-deficient mice, with higher leptin (*Lep*) (Fig. 4l) and lower adiponectin (*Adipoq*) (Fig. 4m) mRNA expression in these mice than in corresponding WT controls.

### Batf3 deficiency favors impaired glucose tolerance, dyslipidemia and liver steatosis in aged mice fed a normal diet

**Fig. 5:**
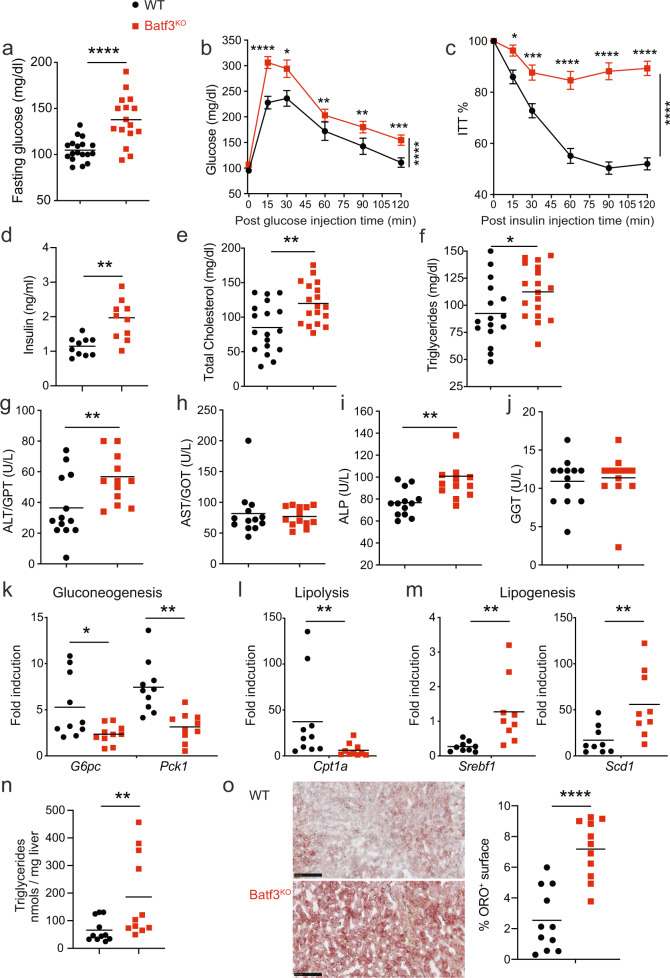
Batf3 deficiency favors impaired glucose tolerance, dyslipidemia and liver steatosis in aged mice fed a standard diet. Glucose levels in serum samples from 30-week-old WT and Batf3^KO^ mice following fasting (**a**) or during a glucose tolerance test (**b**, GTT, *n* = 18). Percentages of baseline glucose levels in mice of the indicated genotypes during an insulin tolerance test (**c**, ITT, *n* = 15). Serum concentrations of basal insulin (**d**), total cholesterol (**e**), triglycerides (**f**), ALT/GPT (**g**), AST/GOT (**h**), ALP (**I**), and GGT (**j**) in WT and Batf3^KO^ 30-week-old mice. Quantification of the liver mRNA expression of **k** gluconeogenesis- (*G6Pc* and *Pck1*), **l** lipolysis- (*Cpt1*a), and **m** lipogenesis- (*Srbf1*, *Scd1*) related genes in mice of the indicated genotypes. Forty- to 50-week-old mice were used for the quantification of triglycerides in the liver (**n**) and quantification of the oil red-positive stained area versus the total liver area in OCR (**o**) liver sections from mice of the indicated genotypes. Bar = 100 μm. Significance was assessed by an unpaired two-tailed Student’s *t*-test, and Welch’s correction was performed in (**n**) and (**o**). *P < 0.05; **P < 0.01; ***P < 0.001; ****P < 0.0001. Each point represents a biological replicate. **b**, **c** Data are presented as the mean ± SEM, and the same mice were measured repeatedly

We investigated whether Batf3-dependent inflammation and altered adipokine production in WAT lead to the metabolic abnormalities that occur during aging. Both obesity and aging can lead to the development of insulin resistance (IR) [31, 32]. Aged mice, like humans, display IR and maintain glucose tolerance through the combination of increased insulin levels, β-cell mass, and β-cell function [33]. No alterations in glucose metabolism were observed in 8-week-old Batf3-deficient male mice (Fig. S3a–c). Batf3-deficient mice exhibited slightly more efficient glucose uptake than WT mice. In contrast, we found increased fasting glucose levels (Fig. 5a) and impaired glucose tolerance (Fig. 5b) in 30-week-old Batf3-deficient mice compared to WT mice. The Batf3-deficient mice were also less sensitive to insulin inoculation (Fig. 5c), indicative of increased insulin resistance, and showed higher basal insulin levels (Fig. 5d). Moreover, we found that fasting glucose levels (Fig. S3d), glucose disposal during a glucose tolerance test (Fig. S3e), and insulin resistance (Fig. S3f) were ameliorated in Batf3^KO^ mice reconstituted with WT BM compared to those adoptively transferred with Batf3-deficient BM.

Batf3 deficiency led to increased lipase activity in the serum (Fig. S3g) and increased total but not free (Fig. S3h) cholesterol (Fig. 5e) and triglycerides (Fig. 5f) in the serum. Since hepatomegaly was evident macroscopically, we determined the serum levels of hepatic enzymes indicative of liver damage. An increase in alanine aminotransferase (ALT, GPT) (Fig. 5g) but not in aspartate aminotransferase (AST, GOT) (Fig. 5h), together with an increase in alkaline phosphatase (ALP) (Fig. 5i) but not in gamma-glutamyl transferase (GGT) (Fig. 5j), in Batf3-deficient mice indicated that these mice could be suffering from nonalcoholic fatty liver disease [34]. Concomitantly, the liver mRNA expression of *glucose 6-phosphatase* (*G6pc*) and *phosphoenolpyruvate carboxykinase* (*Pck1*) (Fig. 5k), enzymes involved in gluconeogenesis, was lower in the absence of Batf3, indicative of liver malfunction. Regarding lipid metabolism, Batf3 deficiency caused decreased *carnitine palmitoyltransferase 1* *A* (*Cpt1a*) expression in the liver (Fig. 5l) concomitant with increased expression of *sterol regulatory element-binding transcription factor 1* (*Srebf1*) and *stearoyl-CoA desaturase* (*Scd1*) (Fig. 5m). These data, together with the finding that Batf3^KO^ mice had an RER > 1 in the dark phase (Fig. 3b), suggest that Batf3 deficiency favors the deposition of fat formed from carbohydrates entering the body. In fact, we observed a higher content of triglycerides in the liver, along with higher lipid deposition in the tissue (Fig. 5n, o).

### Batf3-deficient young mice have relatively few iNKT and NK cells in WAT

Behavioral and metabolic alterations in Batf3^KO^ mice could be a consequence of obesity development, obscuring their causes. Thus, we studied Batf3-deficient mice at 8–9 weeks of life, which is a time point prior to the onset of obesity.

Intestinal homeostasis is dysregulated in mice deficient in IRF8 [35] and in XCR1-DTA mice [36], both of which lack cDC1s. This homeostasis seems to depend on CD103^+^ DCs expressing PDL-1 [37]. We assessed intestinal permeability in Batf3^KO^ mice by administering FITC-dextran via oral gavage and found no differences compared with the permeability of WT mice (Fig. S4a). Our results concur with previous observations suggesting that Batf3 deficiency does not lead to loss of intestinal homeostasis [38], suggesting that higher gut permeability is not the main driver of the obese phenotype observed in Batf3-deficient mice.

At 8 weeks of age, WT and Batf3^KO^ mice showed comparable eWAT weights (Fig. S4b) and similar numbers of CD206^+^ and CD11c^+^ ATMs (Fig. S4c). In contrast to middle-aged mice, 8-week-old Batf3^KO^ mice had decreased proportions and numbers of IFN-γ-producing CD4^+^ T cells compared to WT mice and similar percentages and amounts of CD8^+^ T cells (Fig. S4d, e).

cDC1s contribute to optimal induced regulatory T cells (Treg) priming [39, 40], and WAT contains a population of Treg with a unique phenotype that controls local and systemic inflammation and metabolism [41]. We observed that in the absence of cDC1s, Treg proportions and numbers were increased in eWAT (Fig. S4f-g), particularly those of Tregs expressing GATA3 and IL-33 receptor (ST2) (Fig. S4h and S4i), which are not inducible but rather derived from the thymus.

**Fig. 6:**
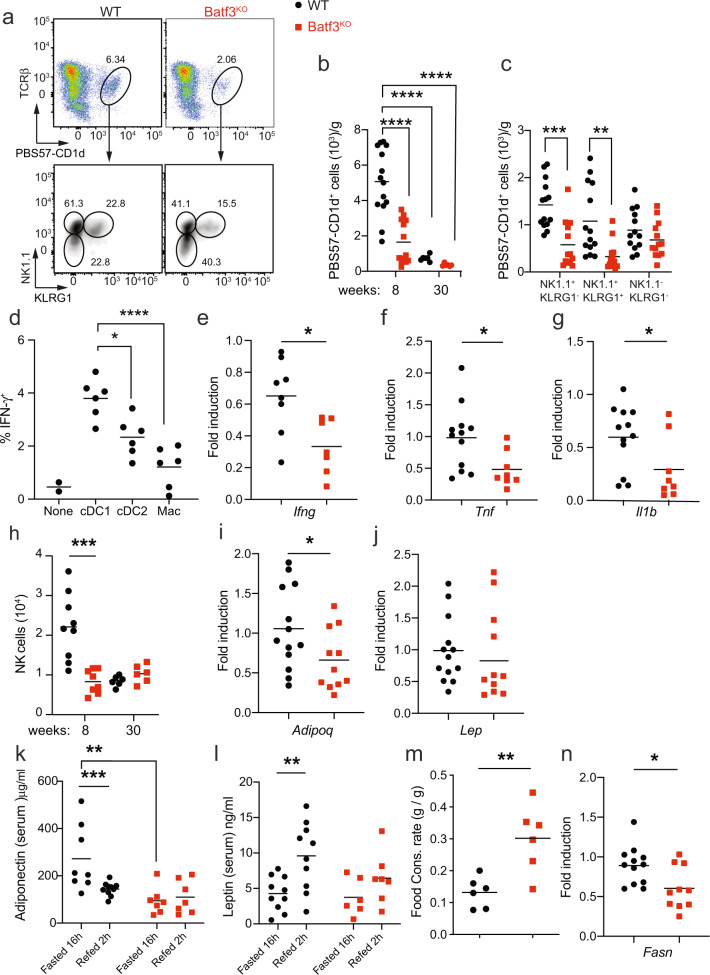
Paucity of iNKT and NK cells in the WAT of young mice deficient in Batf3. Representative flow cytometry dot plots (**a**) and numbers of total iNKT (PBS57-CD1d Tetramer^+^) cells (**b**) and the different subtypes expressing NK1.1 and KLRG1 (**c**) in CD45^+^ cells from the SVF of the eWAT of 8-week-old WT and Batf3^KO^ mice fed an SD. **d** IFN*-γ* production by iNKT cells upon coculture with cDC1s, cDC2,s, or ATMs from the SVF of the eWAT of 8-week-old WT and Batf3^KO^ mice fed an SD. eWAT mRNA expression of *Ifng* (**e**), *Tnf* (**f**), and *Il1b* (**g**). **h** Number of NK cells (CD45^+^ CD3^−^ cells expressing NK1.1, DX5, or both proteins, see Fig. S4j, k) in the eWAT of 8- or 30-week-old mice of the indicated genotypes. Quantification of the eWAT mRNA expression of *Adipoq* (**i**) and *Lep* (**j**) in mice of the indicated genotypes. Serum concentrations of adiponectin (**k**) and leptin (**l**) in mice of the indicated genotype upon overnight fasting and 2 h after refeeding. **m** Quantification of the food consumption rates (g/g) of 8-week-old WT and Batf3^KO^ mice fed an SD. **n** Quantification of the eWAT mRNA expression of *Fasn*. Significance was assessed by an unpaired two-tailed Student’s *t*-test. *P < 0.05; **P < 0.01; ***P < 0.001; ****P < 0.0001. Each point represents a biological replicate

iNKT cells are highly abundant in the visceral adipose tissue of lean mice and humans, where they exert anti-inflammatory functions that contribute to maintaining metabolic homeostasis [42]. Batf3-dependent DCs in the spleen are the most competent presenters of CD1d-associated glycolipids to invariant NKT (iNKT) cells in vivo [43]. Accordingly, we observed low percentages and numbers of iNKT cells in the AT of young Batf3-deficient mice (Fig. 6a, b). Two functional populations of iNKT cells, distinguished by their NK1.1 expression, exist in adipose tissue. IFN-γ produced by NK1.1^+^ iNKT cells is necessary to license NK cells to efficiently remove ATMs [44]. cDC1 deficiency specifically reduced the abundance of NK1.1^+^ iNKT cells (Fig. 6c). Accordingly, we found higher IFN-γ production by iNKT cells sorted from eWAT upon coculture with cDC1s than upon coculture with cDC2s or macrophages (Fig. 6d), while IL-10 production was comparable (Fig. S4i). In addition, the overall eWAT expression of *Ifng, Tnf*, and *Il1b* was decreased in Batf3^KO^ mice compared to WT mice (Fig. 6e–g). This decreased IFN-γ expression correlated with NK cell paucity (Figs. 6h, S4j, k), which could explain the aberrant accumulation of ATMs and increased eWAT inflammation in middle-aged Batf3-deficient mice (Fig. 4).

Young Batf3-deficient mice already showed decreased *Adipoq* mRNA expression in eWAT (Fig. 6i), whereas *Lep* mRNA levels were similar to those in WT mice (Fig. 6j). This suggested that the increased leptin expression in the eWAT of middle-aged knockout mice was simply a consequence of the increased fat deposition. As expected, serum adiponectin in young WT mice decreased upon refeeding after fasting (Fig. 6k), whereas leptin increased (Fig. 6l); in contrast, we could not detect any significant increase or decrease in these adipokines in refed Batf3^KO^ mice. In fact, young Batf3-deficient mice were already eating more than their WT controls (Fig. 6m), which could be at least partially explained by this deregulated adipokine expression [45]. Finally, lipogenesis in eWAT was already decreased in young Batf3^KO^ mice, as shown by reduced *Fasn* expression (Fig. 6n), which might impact adipose tissue expandability [46, 47] and thus further contribute to the metabolic disorder observed in these mice.

### DC expansion induced by administration of systemic sFLT3L mitigates diet-induced obesity, hyperlipidemia, and hepatomegaly

**Fig. 7:**
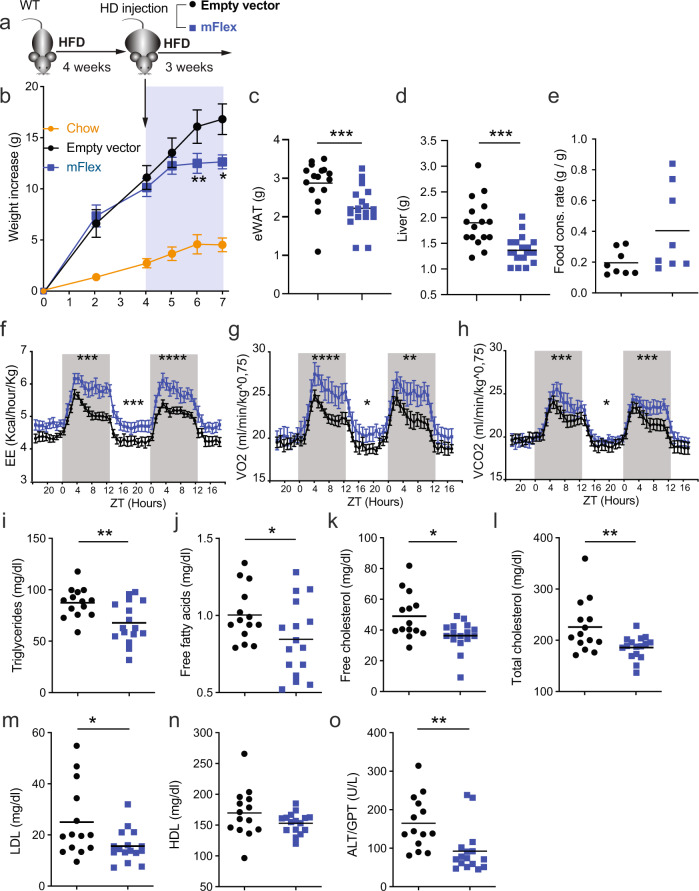
Administration of systemic sFLT3L mitigates DIO, hyperlipidemia, and hepatomegaly. **a** WT mice were fed an HFD for 4 weeks and then treated with a hydrodynamic (HD) injection of a plasmid expressing sFlt3L (mflex). **b** Weight gain (empty vector, *n* = 18; mflex, *n* = 20; SD *n* = 10), eWAT weight (**c**), liver weight (**d**), and quantification of the food consumption rate (**e**) of mice in the indicated groups. Mice treated with mflex or the empty vector were analyzed in metabolic cages for 48 h (*n* = 8); **f** energy expenditure (EE, Kcal/h/kg), (**g**) consumed O_2_ volume (VO_2_), and expired CO_2_ volume (VCO_2_) (**h**), normalized by body weight. The serum concentrations of triglycerides (**i**), free fatty acids (**j**), free cholesterol (k), total cholesterol (l), LDL (m), HDL (n), and ALT/GOT (o) of mice in the indicated groups. Two independent experiments out of three were pooled. Body weight gain quantification (empty vector, *n* = 18; mflex, n = 20; SD *n* = 5). Significance was assessed by an unpaired two-tailed Student’s *t*-test. *P < 0.05; **P < 0.01; ***P < 0.001; ****P < 0.0001. Significance in (f), (g), and (h) was determined by comparing the AUCs of the light and dark periods. Each point represents a biological replicate. (b, f, g, h) Data are presented as the mean ± SEM, and the same mice were measured repeatedly

Given the effects of cDC1 loss on AT homeostasis and weight gain, we investigated whether expansion of conventional DCs could have therapeutic potential in DIO. With this aim, we fed WT mice an HFD, and after 4 weeks of HFD feeding, we administered a hydrodynamic (HD) injection of a plasmid (mFlex) expressing the secreted extracellular region of FLT3L (sFLT3L) (Fig. 7a). Compared to the corresponding control vector, mFlex increased the abundance of cDC1s within the eWAT of mice fed an HFD by 3.5-fold, while the abundance of cDC2s was augmented only 1.7-fold (Fig. S5a, b).

Notably, weight gain was reduced in mice treated with sFLT3L (Fig. 7b). These mice had a lower epididymal fat mass (Fig. 7c), with a reduction in adipocyte size (Fig. S5c), and smaller livers (Fig. 7d) than controls inoculated with the empty vector. These sFLT3L-mediated effects were not due to a reduced food consumption rate (Fig. 7e) or increased activity (Fig. S5d). Whole-body metabolism analysis revealed that HD inoculation of mFlex increased EE (Fig. 7f), increasing the consumed O_2_ volumes mainly in the dark period (Fig. 7g) and expired CO_2_ in the resting period, compared to control treatment (Fig. 7h). Moreover, the administration of sFLT3L reduced the blood levels of triglycerides (Fig. 7i) and free fatty acids (Fig. 7j). Similarly, sFLT3L also decreased free and total cholesterol and LDL (Fig. 7k–m) but not HDL (Fig. 7n). In addition, sFLT3L decreased ALT/GPT in the serum (Fig. 7o) without affecting AST/GOT and GGT (Fig. S5e, f). Taken together, these results suggest that the therapeutic administration of FLT3L may be useful for restraining the onset and development of diet-induced obesity.

### The therapeutic effect of sFLT3L requires Batf3-dependent cDC1s and iNKT cells

**Fig. 8:**
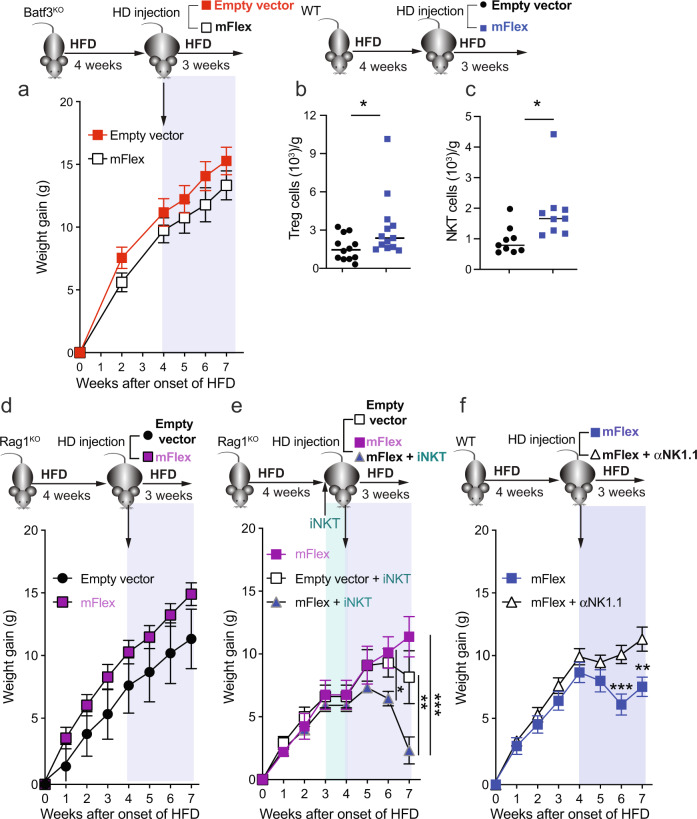
The therapeutic effect of systemic sFLT3L requires Batf3-dependent cDC1s and can be mediated by iNKT cells. Batf3^KO^ (**a**), Rag1^KO^ (**d**, **e**), and WT mice (**b**, **c**, **f**) were fed an HFD for 7  weeks. At week 4, the mice were treated with a hydrodynamic (HD) injection of a plasmid expressing sFlt3L (mflex) or an empty vector, as shown in Fig. 7a. **a** Body weight gain of Batf3^KO^ mice (empty vector *n* = 7; mflex *n* = 9). Quantification of Treg (**b**) and NKT cells (**c**) present in the WAT of WT mice treated with mflex or the empty vector. **d** Body weight gain of Rag^KO^ mice (d, *n* = 9 in both groups). **e** Body weight gain of Rag^KO^ mice with or without adoptive transfer of iNKT cells (*n* = 6 mflex; *n* = 9 Empty vector + iNKT cells; *n* = 9 mflex + iNKT cells). **f** Body weight gain of WT mice treated with or without an anti-NK1.1 depleting antibody (*n* = 9 mflex; *n* = 10 mflex + anti-NK1.1). (**a**, **b**, **c**, **d**, **f**) Significance was assessed by an unpaired two-tailed Student’s *t*-test. **e** Significance was assessed by one-way ANOVA with Sidak’s correction for multiple comparisons *P < 0.05, **P < 0.01; ***P < 0.001. Each point represents a biological replicate. **a**, **d**, **e**, **f** Data are shown as the mean ± SEM, and the same mice were measured repeatedly

Given that sFLT3L administration may impact different immune populations [12, 48], including cDC2s (Fig. S5b), we sought to determine the involvement of Batf3-dependent cDC1s in the therapeutic effect. Notably, sFLT3L administration did not affect body weight gain in Batf3^KO^ mice fed an HFD (Fig. 8a), nor did it change eWAT or liver weight (Fig. S6a, b). Similarly, we did not find any effects on dyslipidemia associated with obesity (Fig. S6c–h) or serum ALT/GPT activity (Fig. S6i).

We observed that systemic sFLT3L increased the abundances of Treg (Fig. 8b) and iNKT cells (Fig. 8c) in WAT. To ascertain whether the therapeutic effect of sFLT3L required mature T cells, we fed Rag1-deficient (Rag1^KO^) mice, which are devoid of mature T and B cells, an HFD, and at week 4, we treated them with an HD injection of mFlex (Fig. 8d). The effect of sFLT3L on weight gain (Fig. 8d) was lost in Rag1^KO^ mice, and no reduction in eWAT or liver weight was observed (Fig. S6j, k). We investigated whether iNKT cells mediate any beneficial effects of sFLT3L in Rag1^KO^ mice. Although we observed similar iNKT numbers in the eWAT of Rag1^KO^ mice inoculated with mFlex or the empty vector (Fig. S6l), adoptive transfer of iNKT cells and mFlex treatment led to less weight gain, less adiposity, and lower circulating triglycerides (Figs. 8e and S6m, n). Accordingly, we also showed that depletion of NK1.1^+^ cells (including NK and NKT cells) (Fig. S6o, p) partially reverted the sFLT3L-mediated effects observed in WT mice (Fig. 8f and S6q, r). Depletion of NK1.1^+^ cells and mFlex treatment led to increased weight gain (Fig. 8f), more adiposity, and higher circulating triglycerides (Fig. S6q, r). Collectively, these data demonstrate that the therapeutic effect of systemic sFLT3L in DIO requires Batf3-dependent cDC1s and that NK1.1^+^ cells, most likely NK1.1^+^ iNKT cells, mediate these effects.

## Discussion

Here, we addressed the role of cDC1s in regulating obesity during overnutrition and aging and uncovered surprising effects on body weight gain and adiposity. While it has been assumed that DCs play a pathogenic role during overnutrition [10], we found that the abundance of cDC1s in visceral adipose tissue was reduced in mice fed an HFD and that reduced expression of cDC1-related genes was characteristic of mice prone to gain more weight (GSE4692). Accordingly, we demonstrated that a deficiency in cDC1s reduced energy expenditure in middle-aged mice and caused adipose tissue inflammation, correlating with impaired glucose tolerance, IR, dyslipidemia, and liver steatosis. Inflammation plays a clear role in metabolic dysfunction associated with obesity and IR [49, 50].

Macrophages and DCs integrate different stimuli to promote homeostasis and tolerance or to trigger inflammation and immunity [51–55], supporting the notion that these cells contribute to surveying organismal metabolism and are implicated in coordinating the IIMR (reviewed in [2]). The IIMR critically allows correct sensing of the balance between energy demands and energy availability. DCs perform much more effective priming of naïve T lymphocytes than macrophages do. In addition, dietary lipids modulate the abundance of DCs within the lymphoid structures in adipose tissue, and DCs may modulate the cross-talk between adipose tissue immunometabolic responses and other lymphoid cells via lipid-derived molecules [56, 57]. Among the three major DC subsets, cDC1s have, at least in mice, an intrinsic capacity to secrete IL-12 and cross‐present antigens via MHC class I, contributing to the priming of IFN-γ-producing CD4^+^ and CD8^+^ T cells [13, 22–24]. All of these functions, along with other signals, enhance macrophage polarization into proinflammatory M1-like cells [20, 21]. Flt3l^KO^ mice devoid of cDCs and pDCs gain less weight than WT mice when fed a high-fat diet (HFD) and have reduced numbers of macrophages in their WAT [10], and several studies have suggested that increased inflammatory effector T cells precede the recruitment of macrophages into adipose tissue [58]. Therefore, we hypothesized that Batf3-dependent cDC1s are critical in triggering obesity. Intriguingly, we observed that Batf3 deficiency mildly increased IR but had no effect on body weight in mice fed an HFD, which might be explained by the reduced abundance of cDC1s in the eWAT of mice suffering DIO. Accordingly, the number of cDC2s (CD1c^+^) but not that of cDC1s (CD141^+^) in subcutaneous fat correlates positively with BMI and IR in humans [59]. This study also identified inflammatory DCs (CD11c^high^F4/80^low^) in the AT of obese mice as Th17 inducers associated with IR [59]. In fact, chronic overnutrition reduces activation of the Wnt/β-Catenin pathway in WAT cDC1s, a pathway that suppresses inflammation through enhanced IL‐10 production, which is an important regulatory mechanism involved in fat expansion [60]. Deletion of β-catenin in the cDCs of mice fed a Western diet decreases adiponectin and reduces glucose tolerance while increasing IR, but SD feeding has no effect on these parameters. In contrast, we found that on an SD, where cDC1 numbers in WAT are conserved, mice deficient in Batf3 displayed low levels of adiponectin in the serum, reduced glucose tolerance, and increased IR but also increased body weight and fat mass as they aged, with reduced energy expenditure and activity and altered oxygen consumption. Our results suggest that Batf3-dependent cDC1s exert anti-inflammatory functions in adipose tissue that are not entirely dependent on the Wnt/β-Catenin pathway [60]. M1-like macrophages accumulate in the WAT of mice deficient in cDC1s, correlating with increased TNF*α* and leptin expression in the tissue, along with lifelong reduced adiponectin expression. Leptin is well known for its ability to regulate food intake and whole-body metabolism, but it is also a proinflammatory cytokine with important effects on immune cell number and function that may partially explain the phenotype of Batf3-deficient mice as they age. However, adiponectin expression was already reduced in young mice before the onset of the phenotype. Adiponectin is also able to exert the functions listed above, but in the opposite sense, and, given our results, is probably a causal agent with at least a partial contribution to the phenotype. It is produced by adipose tissue in response to fasting and is considered to be an anti-inflammatory cytokine. Lower serum levels of adiponectin have been correlated with both obesity and type 2 diabetes in humans [61]. The secretion of adiponectin and leptin by WAT regulates insulin sensitivity, and TNF*α* also promotes IR [62]. Accordingly, we found impaired glucose tolerance and increased IR in Batf3-deficient mice. Moreover, Batf3-deficient mice presented increased dyslipidemia and liver alterations. Before the onset of the obese phenotype, Batf3-deficient mice had decreased infiltration of NK1.1^+^ iNKT and NK cells within their eWAT, consistent with the capacity of cDC1s to induce higher IFN-γ production in sorted iNKT cells from eWAT compared to that of other antigen-presenting cells. This is revealing, as cDC1s are known to produce CXCL9 and CXCL10 to locally recruit CXCR3-expressing cells, such as NK and NKT cells, in the context of antitumor immunity [63]. Thus, Batf3-dependent DCs in eWAT might be competent presenters of glycolipids in association with CD1d to iNKT cells, as previously described for splenic CD8*α*^+^ cDC1s [43]. Indeed, cDC1s are also required for maximal IFN-γ production by iNKT cells in response to *Streptococcus pneumoniae* infection [64]. Notably, IFN-γ produced by NK1.1^+^ iNKT cells is necessary to license NK cells to efficiently eliminate ATMs in vivo, restraining physiological inflammation, and promoting metabolic health [44], which might explain the accumulation of ATMs in Batf3-deficient mice and associated inflammation. Finally, we tested whether DC expansion induced by administration of sFLT3L had an impact on high-fat diet-induced obesity. We found that sFLT3L administration reduced weight gain in a Rag1-dependent manner, ameliorating hepatomegaly and hyperlipidemia. This effect was associated with increased abundances of iNKT cells and Tregs in eWAT, and the therapeutic effect of systemic sFLT3L was partially recovered in Rag1KO mice adoptively transferred with iNKT cells. Moreover, the protective effect was lost in WT mice treated with a depleting antibody specific for NK1.1 and in Batf3-deficient mice. The combination of these outcomes further supports the relevance of the cDC1-iNKT cell axis in controlling adipose tissue homeostasis. In summary, our results reveal that cDC1s play an essential role in preventing the development of obesity during aging, having an impact on the abundance of iNKT and NK cells in eWAT. Our results shed new light on the role of cDC1s in adipose tissue homeostasis and open new avenues for the treatment of obesity through the potentiation of Batf3-dependent cDC1s.

## Materials and methods

### Mice, animal handling, and procedures

This study was designed to determine the roles of Batf3 and cDC1s in obesity. The study was designed for experimental groups including at least four mice per condition. In some cases, experimental sets were repeated under the same conditions but at different times of the year to exclude seasonal variations. Experimental groups were randomly assigned.

The mice used in this study were males unless otherwise indicated. Mice were fed a chow (standard) or high-fat diet beginning when they were 4 or 8 weeks old, respectively, and monitored over 8–10 additional weeks before conducting metabolic studies and end-of-experiment dissection. Additional information can be found in the Reporting Summary. Batf3^KO^ mice were kindly provided by Dr. Kenneth M. Murphy (Washington University, St. Louis, MO, USA) and backcrossed more than ten times with C57BL/6 J mice to establish WT and Batf3^KO^ colonies from the heterozygotes. Rag1^KO^ mice (B6.129S7-Rag1tm1Mom/J) were purchased from The Jackson Laboratory (Bar Harbor, ME, USA). The local ethics committee approved all animal studies. All animal procedures conformed to EU Directive 86/609/EEC and Recommendation 2007/526/EC regarding the protection of animals used for experimental and other scientific purposes, enforced in Spanish law under Real Decreto 1201/2005. The control and treatment groups were assessed at the same time during experiments. Groups were randomly established, and control mice were housed with untreated mice. Mice were never segregated by treatment group.

### Adoptive transfer of syngeneic bone marrow (BM)-derived cells

Mice at 6–8 weeks of age were either sublethally irradiated once (2 Gy) to promote engraftment or lethally irradiated with two doses of 5.5 Gy with a 4 h interval. Adoptive transfer of 3 × 10^6^ cells was performed 24 h after irradiation. The lethally irradiated mice received one dose of cefovecin. Chimerism was directly assessed in the spleen and eWAT after euthanasia.

### Organ harvesting

Mice were starved for 12–16 h and euthanized by intraperitoneal (i.p.) injection of 200 mg/kg sodium pentobarbital (DOLETHAL, Vetoquinol). Prior to harvest of the organ of interest, the mouse heart was perfused with 10 ml of PBS (Life Technologies). eWAT samples were stored at 4 °C in 2 ml of HBSS without calcium or magnesium (Thermo Fisher Scientific) supplemented with 0.5% BSA (Sigma). Liver and eWAT samples were stored at −80 °C in 300 µl of TRIzol (Thermo Fisher Scientific) for RT–qPCR gene expression analysis.

### Cell isolation and flow cytometry analysis

The stromal vascular fraction (SVF) cells in eWAT were isolated as previously described [65]. In brief, after being mechanically disaggregated, eWAT was digested in HBSS supplemented with 0.5% BSA and 10 mM CaCl2 containing 4 mg/ml type II collagenase (Worthington Biochemical Corporation) for 20 min at 37 °C. The digestion was stopped with FBS. The cells were filtered twice through 100 µm and 35 µm cell strainers and centrifuged sequentially at 600 g for 10 min and 5 min. The pellet was diluted in FACS buffer (PBS supplemented with 3% FBS, 2 mM EDTA, and 0.2% sodium azide) before proceeding with antibody staining. Samples for flow cytometry analysis were stained in ice-cold PBS supplemented with 2 mM EDTA, 1% FCS and 0.2% sodium azide. Cells were preincubated for 10 min at 4 °C with anti-mouse CD16/CD32 (clone 2.4G2, Tonbo Bioscience) before staining with the appropriate antibody cocktails. Events were acquired on a FACSCanto or Fortessa flow cytometer (Becton Dickinson), and data were analyzed using FlowJo software (TreeStar). Anti-mouse CD45 eFluor450-conjugated, anti-mouse IL-1 beta Pro-form PE-conjugated, anti-mouse MERTK monoclonal antibody (DS5MMER) Alexa Fluor 700-conjugated, anti-mouse/rat Foxp3 FITC-conjugated, and anti-GATA3 PE-conjugated antibodies were purchased from eBioscience (Thermo Fisher). Anti-mouse CD45.2 Brilliant Violet (BV) 570™-conjugated, anti-mouse CD64 (FcγRI) PerCP-Cy5.5-conjugated, PE-conjugated anti-mouse CD49b (DX5), anti-mouse CD64 PE-conjugated, anti-mouse F4/80 Alexa647-conjugated or biotinylated and APC/Fire™ 750-conjugated, anti-mouse CD11c BV650™-conjugated, FITC- or PE-conjugated anti-mouse CD206, anti-mouse CD9 APC/Fire™ 750-conjugated, anti-mouse IFN-γ APC/Cy7-conjugated, anti-mouse NK1.1 PerCP-conjugated, anti-mouse IL-17-A BV605™-conjugated, anti-mouse CD8a Alexa Fluor 647-conjugated, anti-mouse NK-1.1 Alexa Fluor 700-conjugated, anti-mouse IL-33R*α* (IL1RL1, ST2) PerCP-Cyanine 5.5-conjugated, anti-mouse/rat XCR1 PE-conjugated, anti-mouse TNF-*α* PE-conjugated, anti-mouse/human CD11b BV605-conjugated, anti-mouse CD24 Pe/Cy7-conjugated, PE/Dazzle™ 594-conjugated anti-mouse TCR β chain, and anti-mouse Ly-6C BV711™-conjugated antibodies were obtained from BioLegend. Anti-mouse I-A/I-E (MHC II) FITC-conjugated, anti-mouse CD40 APC-conjugated, anti-mouse CD86 PE-conjugated, anti-mouse iNOS/NOS Type II FITC-conjugated, rat anti-mouse CD90.2 BV786-conjugated, rat anti-mouse CD25 BV711-conjugated, rat anti-mouse CD103 BV421-conjugated, rat anti-mouse IL-10 APC-conjugated, anti-mouse CD11b Brilliant Violet 650- or APC-Cy7-conjugated, anti-mouse XCR1 PE-conjugated, anti-mouse CD90.2, anti-mouse CD4 PE-Cy7-conjugated, anti-mouse CD8 PerCP-Cy5.5-conjugated, anti-mouse CD25 Brilliant Violet 711-conjugated, and anti-mouse NK1.1 PE-conjugated were acquired from BioLegend. An anti-mouse Foxp3 FITC-conjugated antibody was purchased from eBioscience, and a rat anti-mouse CD45R/B220 BV786-conjugated antibody was purchased from BD Biosciences. An APC-conjugated PBS57 (an *α*-GalCer analog)-loaded CD1d tetramer and a PE-conjugated unloaded CD1d tetramer were provided by the NIH tetramer facility (USA). iNKT cells were identified by the expression of TCRβ and staining with the PBS57-loaded CD1d tetramer (compared to the unloaded CD1d tetramer control). For intracellular staining, cells were fixed and permeabilized with a Foxp3/Transcription Factor Staining Buffer Kit (Tonbo) or fixed in 4% PFA and intracellularly stained during permeabilization with 0.1% saponin for intracellular cytokine analysis. Macrophages in eWAT were identified as CD45^+^ CD64^+^ F4/80^+^ cells, CD11c^+^ macrophages were considered proinflammatory M1 macrophages, while those expressing CD206 (mannose receptor) were considered M2 macrophages. The percentage of positive cells was calculated and is indicated within dot plots. Each experiment contained a minimum of three biological replicates, and a minimum of two independent experiments was performed. The percentage and mean fluorescence intensity (MFI) data from sets of experiments are graphed as the mean ± s.e.m.

### Depletion of NK1.1^+^ cells and isolation and coculture of iNKT cells with antigen-presenting cells from WAT

For depletion of NK1.1-expressing cells, mice were i.p. inoculated with 100 μg of anti-NK1.1 depleting antibody (InVivoMAb anti-mouse NK1.1, BioXCell, BE0036) every 3 days.

For adoptive transfer experiments, iNKT cells were isolated from pooled spleens and lymph nodes from WT mice. Cells were first stained with a PE-conjugated anti-NK1.1 antibody and magnetically selected using anti-phycoerythrin (PE) MicroBeads (Miltenyi Bio-tec). Then, NK1.1^+^ TCRβ^+^ cells were sorted, and 2 × 10^5^ cells were i.v. inoculated into mice.

Antigen-presenting cells were magnetically sorted from eWAT single-cell suspensions using a biotinylated anti-MHC II (MHC I-A/I-E) antibody and magnetic streptavidin beads with MACS® columns and autoMACS™ Running Buffer according to the manufacturer’s instructions (Miltenyi Bio-tec). The cells were further sorted into CD11b^+^CD64^+^ macrophages, CD64^–^ XCR1^+^ CD11b^−^ cells and CD64^–^XCR1^−^ CD11b^+^ cells using a FACSAria Sorter. Purified macrophages, cDC1s and cDC2s were cultured in round-bottom 96-well plates (Corning) at 1 × 10^4^ cDC1s/200 μl of R10 medium. Then, iNKT (PBS57-loaded CD1d tetramer^+^) cells (1 × 10^4^) were added to the cultures for 6 h, with brefeldin A (Sigma, 5 μg/ml) added for the last 4 h of culture.

### Serum analysis

Blood samples were obtained by cardiac puncture. The serum was collected after blood centrifugation at 1000 × g for 10 min and frozen at −80 °C. Serum biochemical parameters were analyzed with a Dimension RxL Max automated analyzer the day after the extraction. Urine was collected from mice in the morning. Each urine sample represents a pooled sample from three independent mice. Glucose and protein contents were measured with a Dimension RxL Max automated analyzer.

### Fasting and refeeding

Animals were trained for 1 week by removing their food for 16 h overnight. The last morning, the mice were weighed, and blood was collected from the submandibular vein. Then, the mice were individually caged and given an SD and water for 2 h. Then, submandibular vein blood was collected again.

### Glucose metabolism assessment

Mice were fed a normal standard diet or high-fat diet (HFD) (Research Diets Inc, 60% kcal fat, 1.5% kcal cholesterol) for 8–10 weeks and weighed every week. For the glucose tolerance test (GTT) and insulin tolerance test (ITT), mice were fasted for 16 or 2 h, respectively, with free access to water. For the intraperitoneal (i.p.) GTT, mice received injections of 2 g/kg glucose. For the IP ITT, mice received injections of 0.75 U/kg insulin. The mice were bled from a tail clip, and the blood glucose level was measured with a handheld glucometer before injection (time 0) and at the indicated times after injection. For the insulin resistance (IR) test, mice were injected with 2 g/kg glucose, and blood was collected by a submaxillary puncture at 0, 10, and 30 min after injection. Insulin in the serum was quantified using enzyme-linked immunosorbent assay (ELISA) (Millipore or Thermo Fisher Scientific) following the manufacturer´s instructions.

### Metabolic cages

The energy expenditure (EE), consumed O_2_ volume (VO_2_), expired CO_2_ volume (VCO_2_), respiratory exchange ratio (RER), food, and water intake of mice were quantified using an indirect calorimetry system (TSE LabMaster, TSE Systems, Germany) for at least 2 days after a 2–3 day acclimation period.

### Magnetic resonance imaging

Whole-body and fat imaging of mice was performed with a magnetic resonance scanner. Spectroscopy examinations of WAT were performed in vivo with a 7 T preclinical system (Agilent Varian, Palo Alto, USA) equipped with a DD2 console and an active shielded 205/120 gradient insert coil with 130 mT/m maximum strength. A double-tuned circular transmit/receive coil was used for phosphorus/protons (20 mm), which was placed over the epididymal fat and BAT (Rapid Biomedical GmBH, Rimpar Germany). Images were analyzed with ImageJ software.

### Histopathological staining

Tissue samples were fixed in 4% paraformaldehyde (24 h), processed and embedded in paraffin. Sections (5 μm) were prepared and mounted on slides for staining with hematoxylin and eosin or Masson’s trichrome. Alternatively, liver tissue samples were rehydrated in 30% saccharose for 3 days and embedded in OCT compound (Tissue-Tek). Sections (8 μm) were stained with an oil red stain (0.7% in propylene glycol) for lipid staining. For lipid content quantification, liver slides were digitalized, analyzed with NDP.view2 viewing (Hamamatsu), and quantified with ImageJ software to evaluate the oil red-positive area versus the total area.

### RNA isolation and quantitative PCR

Total RNA was isolated from hepatocytes and adipose tissue using TRIzol (Thermo Fisher Scientific) in combination with an RNeasy Mini Kit (QIAGEN) according to the manufacturer’s instructions. RNA was reverse transcribed into cDNA using random hexamers and a High Capacity cDNA Reverse Transcription Kit (Applied Biosystems). Quantitative PCR amplification was performed with GoTaq qPCR Master Mix (Promega) on a 7900HT Fast Real-Time PCR System (Applied Biosystems). All reactions were performed in triplicate, following the manufacturer’s instructions. Data were normalized to the data for *Gapdh* in each case and are displayed as relative values. Primer sequences are shown in Table S1.

### sFLT3L hydrodynamic injection

For DC expansion, mice were treated with a hydrodynamic (HD) injection of a plasmid expressing sFlt3L (mFlex) (pUMVC3-mFLex, Aldevron). Controls were treated with an HD injection of an empty vector instead (pUMVC3, Aldevron). Inoculation was carried out intravenously (i.v.) via the mouse tail by injecting 10 µg of plasmid diluted in 2 ml of tempered PBS (volume equivalent to 8–12% of the body mass of the mice (21–25 g)) [66, 67].

### Triglyceride quantification

Left liver lobe samples from 40- to 50-week-old mice were weighed and then mechanically disrupted in 5% NP-40. One milliliter of NP-40 was added per 17 mg of the wet liver, and then triglycerides were quantified using a Hepatic Steatosis Kit (Sigma-Aldrich).

### Adipocyte size

The method used for adipocyte size quantification is detailed in [68]. In brief, epididymal adipose tissue was fixed with 4% paraformaldehyde and embedded in paraffin. Tissue sections were stained with hematoxylin and eosin. Using ImageJ, the scale of the image was set in pixels/micra, the image background was subtracted, and the noise in the image was cleaned by despeckling. Then, a threshold was set at the point where only adipocyte membranes were highlighted. The image was then converted to binary, and the dilate tool was used to thin the borders. Then, the wand tracing tool was used to measure the area of all adipocytes that had a complete membrane and were not on the border of the image. The resulting data were then analyzed using GraphPad Prism. Values below 350 μm^2^ or above 15,000 μm^2^ were excluded. Then, the means were binned every 500 μm^2^ and tabulated as relative frequencies. Significance was measured using the Mann–Whitney test.

### Intestinal permeability

Intestinal permeability was assessed in vivo with fluorescein isothiocyanate (FITC)-dextran in two independent experiments, as previously described [69]. Briefly, after 16 h of fasting, mice were orally gavaged with FITC-dextran (Sigma–Aldrich, Madrid, Spain) (5 mg per mouse dissolved in 100 µL of water). After 4 h, all animals were sacrificed, and blood was collected from the heart. Plasma was diluted with PBS, and fluorescence was measured (excitation: 492 nm, emission: 525 nm).

### Statistical analysis

Comparisons among three or more groups were made by one-way or two-way ANOVA. Post hoc corrections for multiple comparisons were made with Tukey’s or Sidak’s test when appropriate. Pairwise comparisons were made with two-tailed Student’s *t*-tests. Student’s *t*-test was also used to analyze the area under the curve (AUC) data. Differences were considered statistically significant at *P* < 0.05. In the figures, asterisks denote statistical significance (∗*P* < 0.05; ∗∗*P* < 0.01; ∗∗∗*P* < 0.001; and ∗∗∗∗*P* < 0.0001). Data were analyzed with GraphPad Prism7. In the figures, each point represents a biological replicate, and if not otherwise indicated, data are given as the mean ± SEM.

## Supplementary information

Supplementary figures

Suppplementary Table

## Data Availability

Further information and requests for resources and reagents should be directed to and will be fulfilled by the lead contact, Salvador Iborra (siborra@ucm.es). This study did not generate new unique reagents. The mouse lines obtained from other laboratories may require a Material Transfer Agreement (MTA) with the providing scientists.
