# Supplementary material for: Conventional type 1 dendritic cells protect against age-related adipose tissue dysfunction and obesity
Source: Cell Mol Immunol. 2022 Jan 4;19(2):260–75. doi: 10.1038/s41423-021-00812-7 (PMC8803960; doi:10.1038/s41423-021-00812-7)
Supplement: Supplementary file 1 — Supplementary figures [file 41423_2021_812_MOESM1_ESM.pdf]

Supplemental Information

Figures S1-S6

Conventional type 1 dendritic cells protect against age-related adipose tissue dysfunction and obesity.

Elena Hernández-García, Francisco J. Cueto, F.J., Emma C. L. Cook, Ana Redondo-Urzainqui, Sara Charro-Zanca, Iñaki Robles-Vera, Ruth Conde, Ivana Nikolić, Guadalupe Sabio, David Sancho and Salvador Iborra.

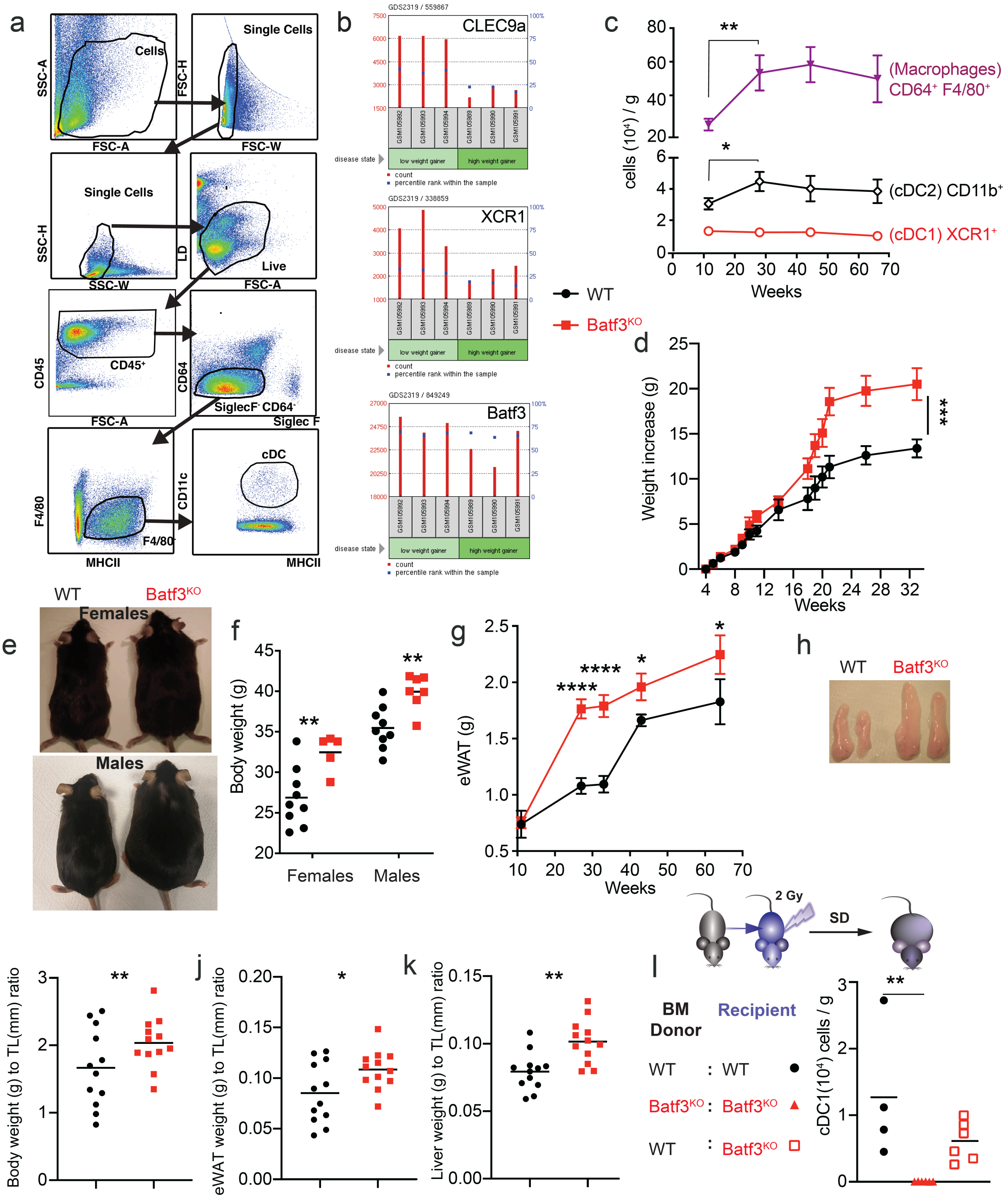

Figure S1. Lack of Batf3 increases body weight gain and adiposity in mice fed a standard chow diet. (a) Gating strategy to identify cDC in eWAT. (b) GEO Profiles Charts depicting the level of expression of *Clec9a*, *XCR1* and *Batf3* genes from epididymal fat of inbred mice that would subsequently become high or low weight gainers after exposure to high fat diet (GSE4692) (c) Quantification of adipose tissue macrophage ($CD64^{+}F4/80^{+}$, ATM) and DC (cDC1 and cDC2) per gram of eWAT at the indicated time points in WT and $Batf3^{KO}$ male mice fed a standard chow diet (SD). (d) Weight gain in

WT and Batf3^{KO} female mice fed a SD for the indicated time points (n=9). Representative images of female and male mice fed a SD at week 30 (e) and body weight (f). (g) Weight of eWAT of WT and Batf3^{KO} mice at the indicated time points. (h) Representative images of eWAT pads of the indicated genotypes. Weights normalized to tibia length (mm) of body (i), eWAT (j) and liver (k) of mice at 70 weeks of age. (l) WT mice were irradiated and adoptively transferred with WT BM cells, or Batf3-deficient recipients adoptively transferred with Batf3-deficient or WT BM cells. Quantification of cDC1 in the epididymal White Adipose Tissue (eWAT) was determined by flow cytometry 24 weeks after irradiation. Significance assessed by unpaired two-tailed Student's t test. Student's t test was also used to analyze area under curve (AUC) data (d) *, $P<0.05$; **, $P<0.01$; ***, $P<0.001$; ****, $P<0.0001$. Each point represents a biological replicate. (c, d, g) Data are the mean ± SEM.

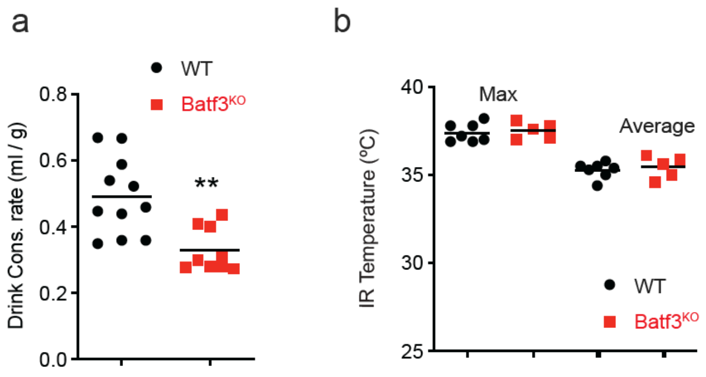

Figure S2. Water intake and body temperature in Batf3-deficient mice. (a) Drink consumption of 30-week-old WT and Batf3^{KO} mice analyzed in metabolic cages for 48 hours. (b) Skin temperature of surrounding interscapular BAT determined by thermographic images. Significance assessed by unpaired two-tailed Student's t test. **, *P*<0.01. Each point represents a biological replicate, bars represent the mean.

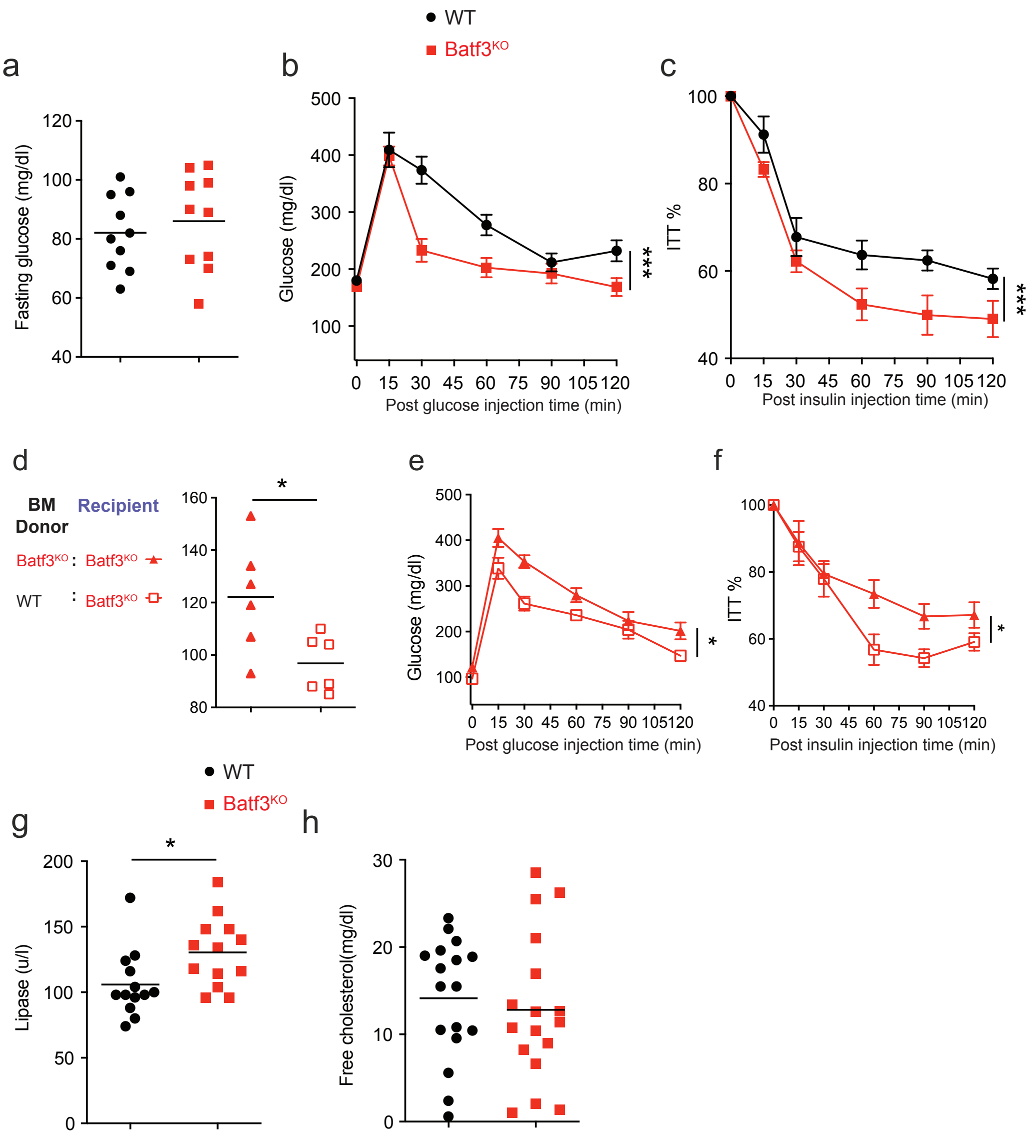

Figure S3. Impaired glucose metabolism in Batf3-deficient mice. Glucose levels in serum samples in WT and Batf3^{KO} 8-week-old mice following: (a) fasting or (b) during a glucose tolerance test (GTT, n=10). (c) Percentage of initial glucose levels in mice of the indicated phenotype during an insulin tolerance test (ITT, n=10). Glucose levels in serum samples in 30-week old Batf3^{KO} mice adoptively transferred with WT or Batf3-deficient BM cells (related to figure 2h) , following: (d) fasting or (e) during a glucose tolerance test (GTT, n=6). (f) Percentage of initial glucose levels in mice of the indicated phenotype during an insulin tolerance test (ITT, n=6). (g) Lipase activity and (h) free cholesterol in serum samples of WT and Batf3 KO 30 week-old mice (related to figure 5). Significance assessed by unpaired two-tailed Student's t test of AUC values (b,c,e,f). *, $P<0.05$; ***, $P<0.001$. Each point represents a biological replicate. (b,c,e,f) Data represent the mean ± SEM.

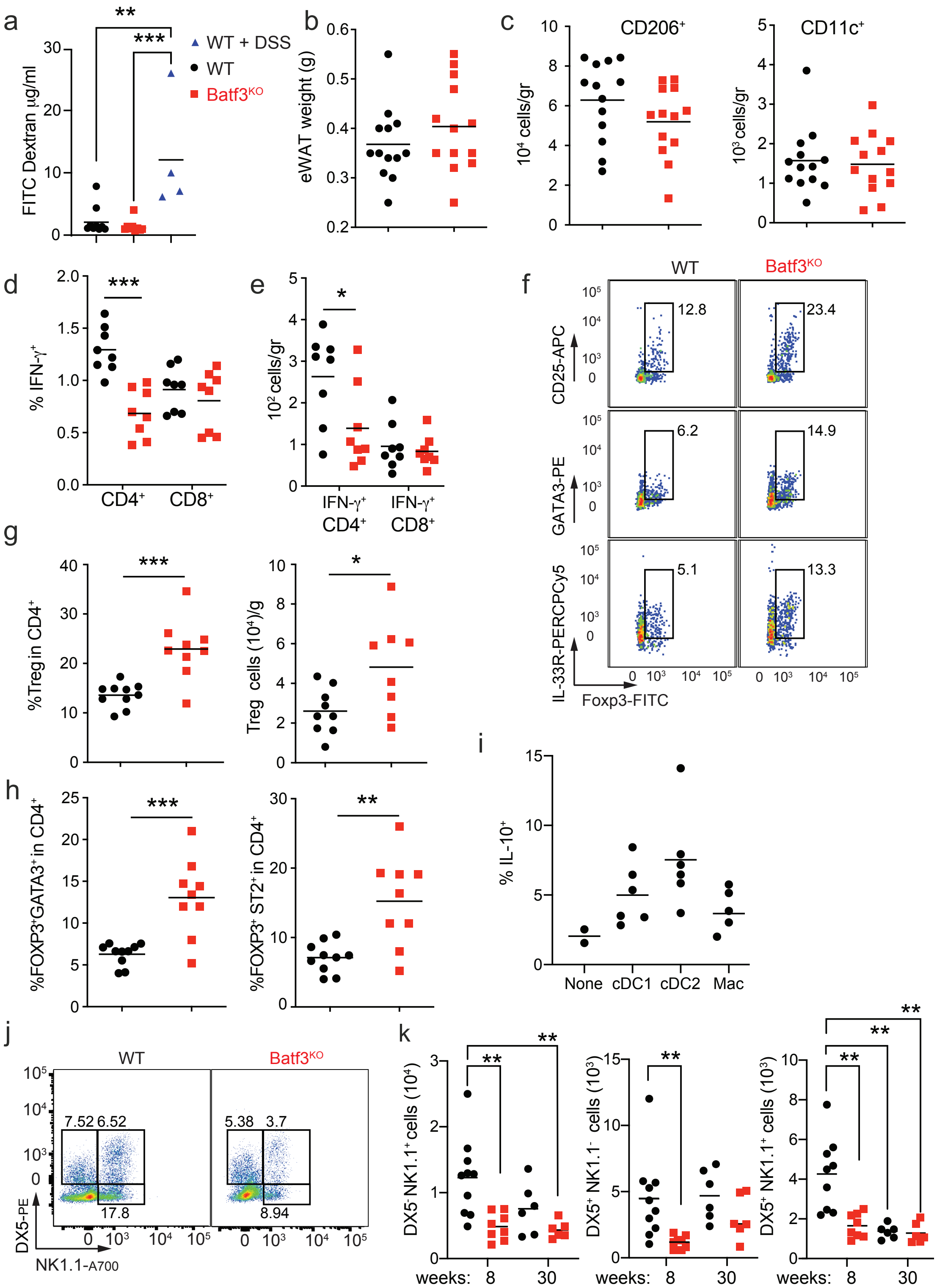

Figure S4. Gut permeability and cellular composition of eWAT of young Batf3-deficient mice fed a normal diet. (a) Mice of the indicated genotypes were fasted for 16h, and then inoculated with FITC Dextran (5mg/mouse). Fluorescence in serum was determined 4h later. DSS-treated WT mice were included as controls. (b) eWAT weight of WT and Batf3^{KO} 8-week-old mice fed a SD. (c) Amount of CD206^{+} and CD11c ATM

per gram of eWAT. Percentages (d) and numbers (e) of IFN-γ producing $CD4^+$ and $CD8^+$ T cells upon in vitro stimulation of the SVF of eWAT from mice of the indicated genotypes. (f) Representative flow cytometry dot plots of Tregs expressing FoxP3, CD25, GATA3 and ST2 (IL-33R). (g) Percentages and quantification of total Treg in eWAT of the indicated genotypes. (h) Percentages of $GATA3^+$ and $ST2^+$ Treg in eWAT of the indicated genotypes. (i) IL-10 production by iNKT upon culture with cDC1, cDC2 and ATMs from SVF of the eWAT of 8-week-old WT and $Batf3^{KO}$ mice fed a SD as in figure 5C. Representative flow cytometry dot plots (j) of NK cells ($CD45^+$ $CD3^-$) in eWAT, and quantification (k) of the different subpopulations ($DX5^-$ $NK1.1^+$; $DX5^+$ $NK1.1^-$ and $DX5^+$ $NK1.1^+$). Significance assessed by unpaired two-tailed Student's t test. *, P<0.05; **, P<0.01; ***, P<0.001. Each point represents a biological replicate, bars represent the mean.

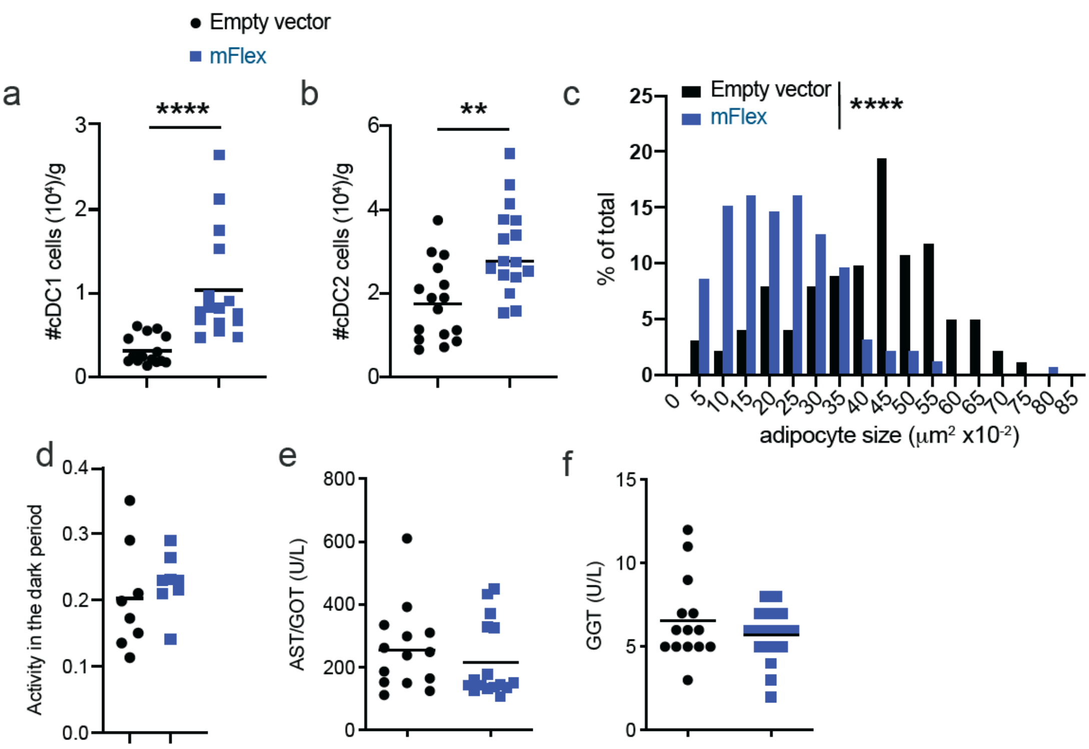

Figure S5. Administration of systemic sFLT3L expands cDC, reduces the adipocyte size, but does not affect activity, AST or GGT. Quantification of adipose tissue cDC1 (a) and cDC2 (b) per gram of eWAT in WT male mice two weeks after HD inoculation of mFlex or empty vector. (c) Frequency distribution of adipocyte sizes in the eWAT of mice treated with mFlex or empty vector. (d) Activity of mice treated or not with mFlex during the dark period. Serum concentrations of (e) AST/GOT, and (f) GGT in WT mice treated as in Figure 7a. Significance assessed by unpaired two-tailed Student's t test (a,b) or Mann-Whitney test (c). **, *P*<0.01; ****, *P*<0.0001. Each point represents a biological replicate, bars represent the mean.

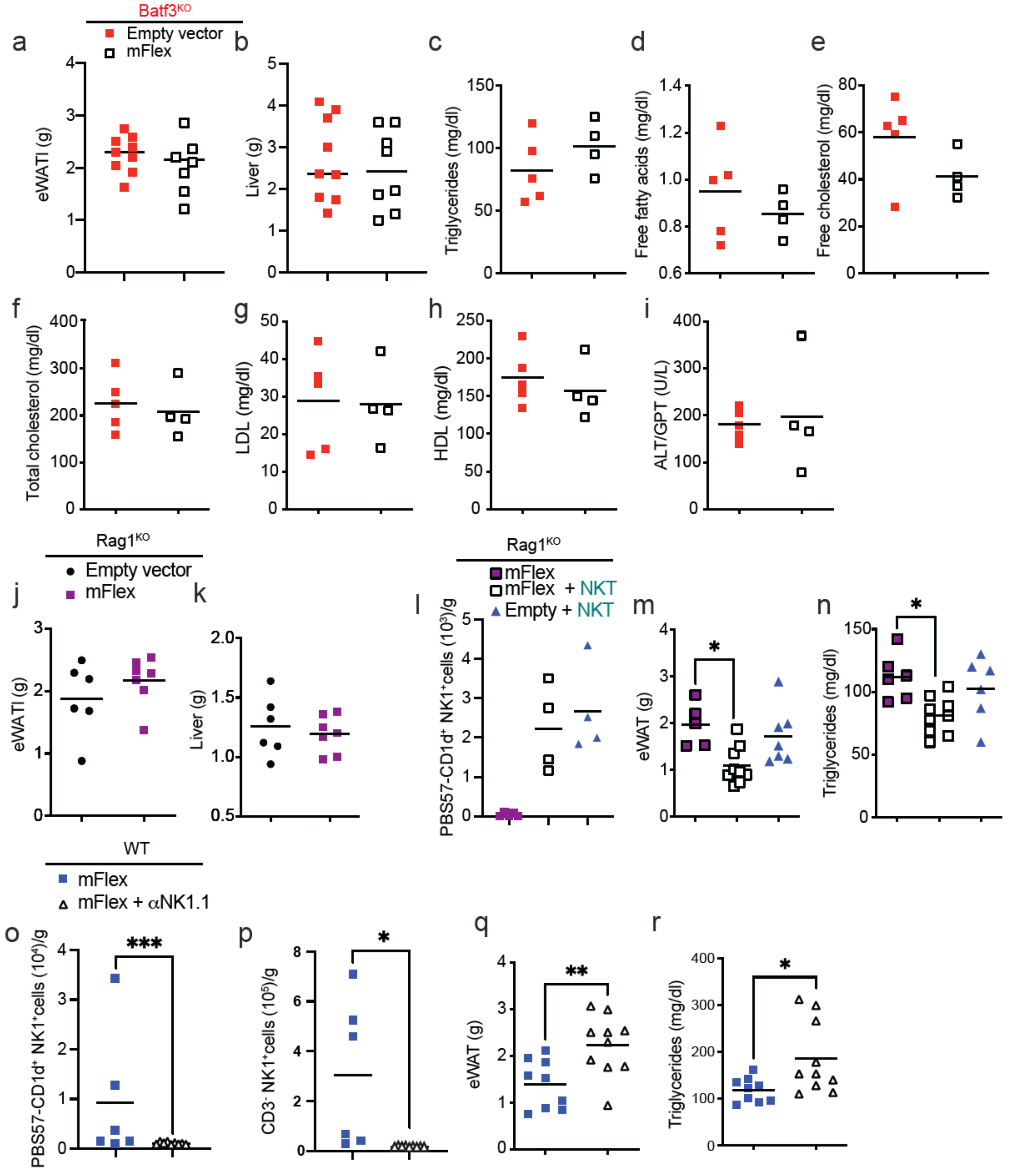

Figure S6. Systemic sFLT3L treatment in HFD-fed mice deficient for Batf3 or Rag1. Mice were fed a HFD during 7 weeks and treated with a hydrodynamic (HD) injection of a plasmid expressing sFlt3L (mFlex) on week 4. Quantification of eWAT (a) and liver (b) weight, serum concentrations of triglycerides (c), free fatty acids (d), free cholesterol (e), total cholesterol (f), LDL (g), HDL (h) and ALT/GPT (i) of Batf3^{KO} mice of the indicated groups (related to figure 8a). Quantification of eWAT (j) and liver (k) weight

of Rag1^{KO} mice of the indicated groups (related to figure 8d). Quantification of eWAT iNKT numbers (l) and weight (m), and triglyceride concentration in serum (r) of Rag1^{KO} reconstituted with WT iNKT (related to figure 8e). Quantification of eWAT cells expressing NK1.1 (o and p), eWAT weight (q) and triglyceride concentration in blood (r) of mFlex-inoculated WT mice, treated or not with an anti-NK1.1 depleting antibody (related to Figure 8f). Significance assessed by unpaired two-tailed Student's t test. Each point represents a biological replicate, bars represent the mean.
