# Supplementary material for: Conventional type 1 dendritic cells protect against age-related adipose tissue dysfunction and obesity
Source: Cell Mol Immunol. 2022 Jan 4;19(2):260–75. doi: 10.1038/s41423-021-00812-7 (PMC8803960; doi:10.1038/s41423-021-00812-7)
Supplement: Supplementary file 2 — Suppplementary Table [file 41423_2021_812_MOESM2_ESM.docx]

| Table S1: Oligonucleotides |
| --- |
| Mouse *Gadph* FWD*:* TGAAGCAGGCATCTGAGGG |
| Mouse *Gadph* REV: CAGGAAGTAGGTGAGGGCTTG |
| Mouse *Il6* FWD*:* CCGTGTGGTTACATCTACCCT |
| Mouse *Il6* REV: CGTGGTTCTGTTGATGACAGT |
| Mouse *TNFa* FWD*:* CCCTCACACTCAGATCATCTTCT |
| Mouse *TNFa* REV: GCTACGACGTGGGCTACAG |
| Mouse *IL-10* FWD: GCTCTTACTGACTGGCATGAG |
| Mouse *IL-10* REV: CGCAGCTCTAGGAGCATGTG |
| Mouse *Leptin* FWD*:* GAGACCCCTGTGTCGGTTC |
| Mouse *Leptin* REV: CTGCGTGTGTGAAATGTCATT |
| Mouse *Adiponectin* FWD:TGTTCCTCTTAATCCTGCCCA |
| Mouse *Adiponectin* REV: CCAACCTGCACAAGTTCCCTT |
| Mouse *Acc* FWD*:* GATGAACCATCTCCGTTGGC |
| Mouse *Acc* REV: GACCCAATTATGAATCGTG |
| Mouse *Ppara* FWD: ACAAGGCCTCAGGGTACCA |
| Mouse *Ppara* REV: GCCGAAAGAAGCCCTTACA |
| Mouse *Fasn* FWD*:* GCGGGTTCGTGAAACTGATAA |
| Mouse *Fasn* REV: GCAAAATGGGCCTCCTTGATA |
| Mouse *Scd1* FWD: TTCTTGCGATACACTCTGGTGC |
| Mouse *Scd1* REV: CGGGATTGAATGTTCTTGTCGT |
| Mouse *Srebpc* FWD*:* GGCACTGAAGCAAAGCTGAA |
| Mouse *Srebpc* REV: TCATGCCCTCCATAGACACA |
| Mouse *Pepck* FWD: CCATCACCTCCTGGAAGAACA |
| Mouse *Pepck* REV: ACCCTCAATGGGTACTCCTTCTG |
| Mouse *Acc* FWD*: GATGAACCATCTCCGTTGGC* |
| Mouse *Acc* REV: GACCCAATTATGAATCGTG |
| Mouse  *Ppara* FWD: ACAAGGCCTCAGGGTACCA |
| Mouse *Ppara* REV: GCCGAAAGAAGCCCTTACA |
| Mouse *Cpt1a* FWD*:* CTCCGCCTGAGCCATGAAG |
| Mouse *Cpt1a* REV: CACCAGTGATGATGCCATTCT |
| Mouse *G6pase* FWD: CGACTCGCTATCTCCAAGTGA |
| Mouse *G6pase* REV: GTTGAACCAGTCTCCGACCA |
| Mouse IL-1b FWD*:* CTGAACTCAACTGTGAAATGCCA |
| Mouse IL-1b REV: AAAGGTTTGGAAGCAGCCCT |
| Mouse IFNγ FWD: CTGCCACGGCACAGTCATTG |
| Mouse IFNγ REV: TGCATCCTTTTTCGCCTTGC |
